# Estimating reassortment rates in co-circulating Eurasian swine influenza viruses

**DOI:** 10.1099/vir.0.044503-0

**Published:** 2012-11

**Authors:** S. J. Lycett, G. Baillie, E. Coulter, S. Bhatt, P. Kellam, J. W. McCauley, J. L. N. Wood, I. H. Brown, O. G. Pybus, A. J. Leigh Brown, for the Combating Swine Influenza Initiative (COSI) Consortium

**Affiliations:** 1 Institute of Evolutionary Biology, University of Edinburgh, Kings Buildings, West Mains Road, Edinburgh EH9 3JT, UK; 2 Wellcome Trust Sanger Institute, Wellcome Trust Genome Campus, Hinxton, Cambridge CB10 1SA, UK; 3 Department of Zoology, University of Oxford, The Tinbergen Building, South Parks Road, Oxford OX1 3PS, UK; 4 Division of Virology, MRC National Institute for Medical Research, Mill Hill, London NW7 1AA, UK; 5 Cambridge Infectious Diseases Consortium, Department of Veterinary Medicine, University of Cambridge, Madingley Road, Cambridge CB3 0ES, UK; 6 Animal Health and Veterinary Laboratories Agency – Weybridge, Addlestone, Surrey, KT15 3NB, UK

## Abstract

Swine have often been considered as a mixing vessel for different influenza strains. In order to assess their role in more detail, we undertook a retrospective sequencing study to detect and characterize the reassortants present in European swine and to estimate the rate of reassortment between H1N1, H1N2 and H3N2 subtypes with Eurasian (avian-like) internal protein-coding segments. We analysed 69 newly obtained whole genome sequences of subtypes H1N1–H3N2 from swine influenza viruses sampled between 1982 and 2008, using Illumina and 454 platforms. Analyses of these genomes, together with previously published genomes, revealed a large monophyletic clade of Eurasian swine-lineage polymerase segments containing H1N1, H1N2 and H3N2 subtypes. We subsequently examined reassortments between the haemagglutinin and neuraminidase segments and estimated the reassortment rates between lineages using a recently developed evolutionary analysis method. High rates of reassortment between H1N2 and H1N1 Eurasian swine lineages were detected in European strains, with an average of one reassortment every 2–3 years. This rapid reassortment results from co-circulating lineages in swine, and in consequence we should expect further reassortments between currently circulating swine strains and the recent swine-origin H1N1v pandemic strain.

## Introduction

The major reservoir for influenza A viruses comprises avian hosts, primarily waterfowl, from which certain strains occasionally cross the species barrier to infect mammals, including swine, humans and horses (Webster *et al.*, 1992). These events can lead to epizootic and epidemic outbreaks, and establishment of new viral lineages in the recipient host populations. Influenza A viruses contain eight RNA segments. These encode the polymerases (PB2, PB1, PA) and nucleoprotein (NP) (segments 1, 2, 3 and 5, respectively); non-structural proteins (NS) (segment 8); matrix protein (MP) and the M2 ion channel protein (segment 7); and the surface glycoproteins, haemagglutinin (HA, segment 4) and neuraminidase (NA, segment 6). The latter two segments define the subtype of the virus. Seventeen subtypes of HA (subtypes H1–H16 in birds and H17 in bats; Tong *et al.*, 2012) and nine subtypes of NA have been observed but, to date, only viruses with HA subtypes H1, H2, H3 and NA subtypes N1 and N2 are known to have established lineages in the human population lasting longer than 1 year. One important factor is that, in humans, HA generally preferentially binds to the α-2,6-linked sialic acid receptors present on respiratory epithelium, whereas HA in avian viruses binds to α-2,3-linked sialic acid receptors (Connor *et al.*, 1994; Matrosovich *et al.*, 2000; Rogers & D’Souza, 1989), although there is variation between viruses and variation in receptor distribution at different levels of the respiratory tract (van Riel *et al.*, 2007, 2010). However, both types of receptor are relatively common in swine trachea, making swine susceptible to infection with both avian and human influenza viruses; consequently, pigs have been suggested to play a role in facilitating the adaptation of avian viruses to human hosts (Ito *et al.*, 1998; Scholtissek *et al.*, 1985).

Phylogenetic analyses have shown that there are distinct lineages of influenza A segments broadly corresponding to host type (Webster *et al.*, 1992), but there have been various cross-species transmissions and reassortments among segments, giving rise to new genotypes or clades (e.g. dos Reis *et al.*, 2009; Dunham *et al.*, 2009; Schultz *et al.*, 1991; Smith *et al.*, 2009). These events have included: (i) H1N1 avian to swine transmission in the 1970s, creating the H1N1 Eurasian swine lineage clade found predominantly in Europe and Asia; (ii) reassortment between avian-like H1N1 and human viruses, resulting in the circulation of H1N2 and H3N2 reassortants in swine in Europe; (iii) the complex H1N1, H1N2 and H3N2 reassortment of avian, human and swine viruses, creating the swine triple-reassortant viruses seen in swine predominantly in North America from the 1990s onwards; and (iv) reassortment between the triple-reassortant and H1N1 Eurasian swine influenza viruses (i) and (iii), creating the 2009 H1N1v human pandemic strain (Brown *et al.*, 1998; Castrucci *et al.*, 1993; Garten *et al.*, 2009; Olsen, 2002; Pensaert *et al.*, 1981; Smith *et al.*, 2009; Webby *et al.*, 2000; Zhou *et al.*, 1999).

Early human cases attributed to the 2009 H1N1v pandemic strain were recorded in late March 2009 [Novel Swine-Origin Influenza A (H1N1) Virus Investigation Team, 2009], and the date of first emergence into the human population was estimated to be around January 2009 (Fraser *et al.*, 2009). However, the common ancestors of the pandemic strain and its Eurasian and triple-reassortant swine precursors existed 10–20 years prior to this, which leaves at least 10 years of unsampled evolutionary history (Smith *et al.*, 2009). This gap has prompted recent studies by Vijaykrishna *et al.* (2011) and Nelson *et al.* (2011), who analysed sequences from swine strains isolated from Hong Kong and China, and North America, respectively, during this approximate time period.

The aims of the current study were to extend this work and to characterize the strains present in European swine by sequencing archived material from 1982 to 2008, in order to show how co-circulation of strains can lead to the generation of reassortants and to estimate the rate of virus reassortment in European swine.

## Results

### Distribution of genotypes

Over 600 virus isolates of swine influenza collected as part of routine surveillance from 1980 onwards at the Animal Health and Veterinary Laboratories Agency (AHVLA) – Weybridge, UK, the WHO Collaborating Centre for Reference and Research on Influenza, Mill Hill, London, UK, and through the European Surveillance Network for Influenza in Pigs (ESNIP) (co-ordinated by Ghent University, Belgium) were available for sequencing. The isolates in the archives had been characterized serologically prior to sequencing. Two isolates per subtype, per location (region or country), per year were selected for sequencing at random from the archives. In the cases where there was only one sample available in a category, we included a sample of the same subtype and year from a different location, where possible.

We report here 28 newly obtained, high-quality complete genome sequences and 18 high-quality partial genome sequences (GenBank accession numbers CY115870–CY116570; see Table S1, available in JGV Online). We combined these with complete genome sequences from 41 H1N1 avian-like Eurasian swine isolates reported in another study by our consortium (Bhatt *et al*., 2012), to make a total of 69 complete genome sequences taken from Belgium, the Czech Republic, France, Italy, Poland, Spain and the UK (see Tables 1 and S2).

**Table 1.** Sequences by host species and subtype used in the initial analysis

Host	H1N1	H1N2	H2N2	H3N1	H3N2	Total
Avian	30	2	6	0	16	54
Human	86	2	21	0	114	223
Swine	62	17	0	0	23	102
COSI swine complete	46	16	0	0	7	69
COSI swine partial	6	6	0	2	4	18

In order to classify each new swine isolate, we examined maximum-likelihood trees generated after combining these data with a selection of complete genome background sequences from the NCBI Influenza Virus Resource (Bao *et al.*, 2008) on a segment-by-segment basis (separate trees were generated for HA and NA subtypes). The sequences were classified as Eurasian swine, triple-reassortant swine, human seasonal-origin, classical swine, avian Eurasian and avian North American (see Figs S1–S10, available in JGV Online, for these trees). As well as the 41 H1N1 Eurasian swine lineage complete genomes (Bhatt *et al*., 2012), we obtained 16 complete genomes of H1N2 swine lineage (i.e. human seasonal origin HA-H1 and NA-N2 and internal protein-coding segments from the Eurasian swine lineage). We also sequenced a reassortant H1N1 isolate from Italy in 2001 and obtained a complete genome in which the Eurasian H1 was replaced by a human seasonal-origin H1 from an H1N2 Eurasian swine strain.

Seven complete genome H3N2 viruses were sequenced. Six of the H3N2 complete genomes contained human seasonal-origin HA-H3 and NA-N2, and Eurasian clade internal gene segments; but one complete H3N2 (and one partial genome) from the UK contained entirely human-origin segments.

The two H3N1 viruses which were sequenced (collected in England) gave partial genomes including segments 3–8 (Figs S3–S10). One isolated in 1992 [A/swine/England/203759/1992(H3N1)] was most closely related to an H3N2 strain from 1993 [A/swine/England/285393/1993(H3N2)] in segments 4, 7 and 8, but to H1N1 Eurasian swine isolates from England 1992–1993 in segments 3 and 6, and to the completely human-origin H3N2 English swine isolates in segment 5 (NP). The other H3N1 isolate from 1995 [A/swine/England/704653/1995(H3N1)] was not a direct descendant of the earlier H3N1 strain, but was very similar to a contemporary H1N1 Eurasian swine strain [A/swine/England/745969/1995(H1N1)] in all its sequenced segments except segment 4 (HA), where it was most similar to an earlier H3N2 swine strain [A/swine/England/375017/1993(H3N2)], implying that only the HA segment had been substituted for a human seasonal-origin H3 (probably originating from a Eurasian H3N2 swine virus; Fig. S5).

Four H1N1 classical swine complete genomes were obtained from UK isolates (1986–1993), but we did not detect any H3N2 triple-reassortant swine viruses in Europe, nor did we find any viruses with the H1N1v pandemic segment constellation prior to the onset of the pandemic in 2009.

### Eurasian swine clade

**Fig. 1.**
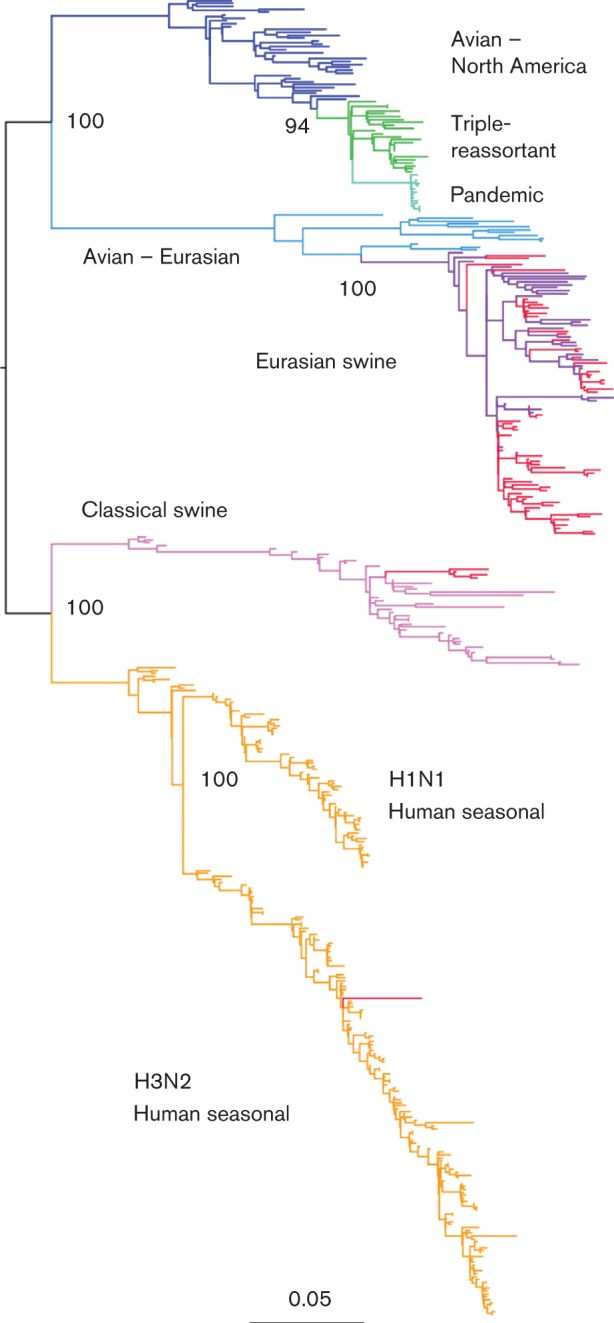
Maximum-likelihood tree for PB2 sequences. The branches of the tree have been coloured according to clade: human seasonal, orange; classical swine, pink; avian Eurasian, pale blue; Eurasian swine, purple; avian North America, dark blue; triple-reassortant swine, green; H1N1v pandemic, turquoise. Swine sequences generated as part of this study are coloured red, and the bootstrap support for selected nodes is shown. Bar, 0.05 nucleotide substitutions per site.

Phylogenies from isolates with complete genomes and the background sequence set revealed a highly supported monophyletic clade (boostrap support ≥99) containing 92 Eurasian swine isolates from Europe (*n* = 81) and Asia (*n* = 11) in segments 1, 2 and 3 (PB2, PB1, PA). A simplified phylogeny for segment 1 is shown in Fig. 1 (see Figs S1–S20 for more detailed maximum-likelihood and beast trees). This clade of 92 Eurasian sequences in segments 1, 2 and 3 was mostly conserved in segments 5 (NP) and 8 (NS); however, two segment 5 sequences from viruses from Asia (in the background set) clustered with the classical swine clade. Also, three segment 8 sequences of viruses from Asia clustered with the classical swine clade, and another Asian segment 8 sequence clustered with the triple-reassortant clade (which descends from the classical swine clade in this segment). On segment 7 (MP) all 92 sequences examined from viruses from Eurasia belonged to the Eurasian lineage, but in addition this clade also contains the pandemic sequences, and a recent reassortant swine sequence from Guangdong (A/Swine/Guangdong/1/2010) (Xu *et al.*, 2011). With exception of the reassortant sequences mentioned, the Eurasian swine clade was highly supported in all internal protein-coding segments (bootstrap values ≥99 for maximum-likelihood trees and posterior probability >0.999 for beast trees).

**Fig. 2.**
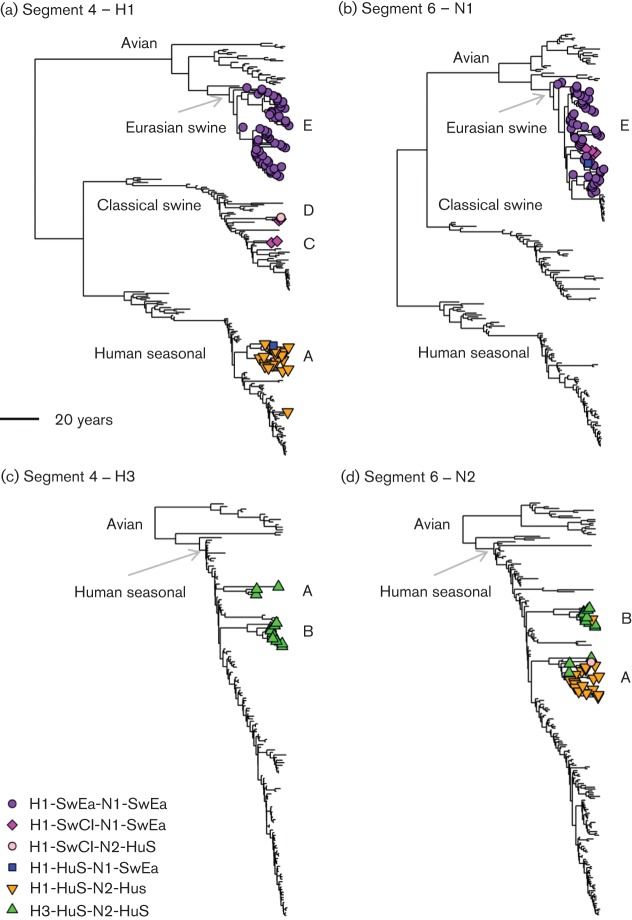
Time-resolved trees for HA-H1, HA-H3, NA-N1 and NA-N2. Marked isolates are all from swine and have Eurasian swine clade segment 1 sequences. (a) Segment 4 – H1; (b) segment 6 – N1; (c) segment 4 – H3; (d) segment 6 – N2. The detailed subtype abbreviations are: SwEa, Eurasian swine; SwCl, classical swine; HuS, human seasonal-origin. The marked clades represent: A, H1N2+H3N2 introduction; B, H3N2 introduction; C, classical swine introduction; D, classical swine introduction; E, Eurasian swine clade.

The dates of the most recent common ancestor (TMRCA) of the Eurasian swine clade were estimated from the beast trees for each internal protein-coding segment, and all estimates were similar (around 1980, confidence intervals ranging between 1975 and 1985) and consistent with previous work (Brown, 2000; Dunham *et al.*, 2009; Scholtissek *et al.*, 1983). However, there were also a number of subsequent introductions of different HA and NA lineage segments which reassorted with the prevailing Eurasian swine clade polymerases. Fig. 2 shows outline HA-H1, HA-H3, NA-N1 and NA-N2 beast phylogenies with the identified 92 isolates marked. The isolates which have Eurasian clade PB2, HA-H1 and NA-N1 are marked as purple circles (clade E). The subsequent reassortments with the Eurasian swine clade polymerases are shown with other markers including reassortments with human seasonal lineages (H1N2, orange triangles; H3N2, green triangles) and the classical swine lineage (dark pink diamonds).

**Fig. 3.**
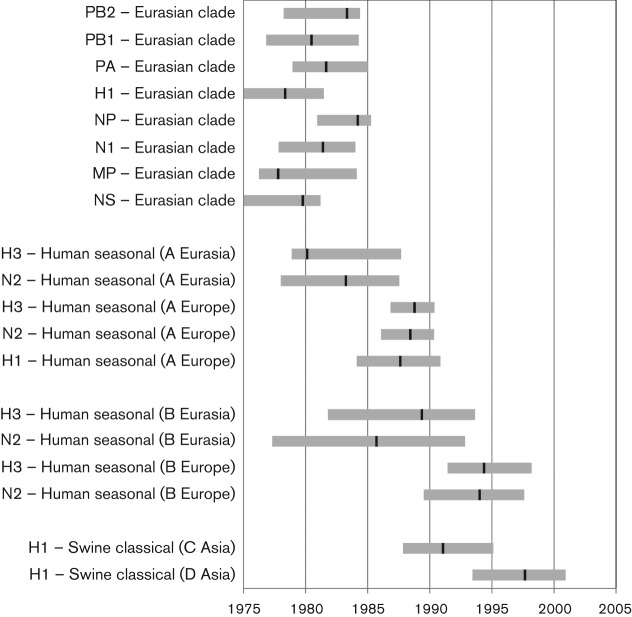
Estimated TMRCA for the Eurasian swine clade per segment, and for the subsequent H1, H3 and N2 introductions to Eurasia and Europe. The letter designation for the subsequent introductions (e.g. A Eurasia) corresponds to the marked clades in Fig. 2. There are only classical swine H1 introductions into Asia (none into Europe) in this dataset. The black bars represent the node age estimates from the beast trees and the grey bars indicate the 95 % HPD range.

Fig. 3 shows the timescale of the formation and subsequent introductions into the Eurasian swine clade as estimated from the ages of the internal nodes of the beast trees for HA and NA from Fig. 2 (see also Figs S11–20). After the initial formation of the Eurasian swine clade, there were two subsequent introductions of human seasonal lineage HA-H1 [introduction A, and the single 2009 sequence in Fig. 2(a)] and two of classical swine lineage HA-H1 [introductions C and D in Fig. 2(a)]. There were also two HA-H3 and two NA-N2 human seasonal lineage introductions [introductions A and B in Fig. 2(c, d)].

One NA-N2 introduction [A in Fig. 2(d)] was predominantly H1N2 (18 out of 21 genomes) into England (13), Continental Europe (six) and Asia (two), with an estimated median TMRCA of 1983. The other NA-N2 introduction [B in Fig. 2(d)] was estimated to occur at a similar time, with a median TMRCA of 1986, but was predominantly H3N2 (10 out of 11 genomes) into Continental Europe (nine) and Asia (two). For both of these introductions, we also estimated the TMRCA of the European sequences only, since these formed separate subclades from the Asian isolates. The first predominantly H1N2 introduction into Europe (A) had a median TMRCA of 1988–1989 (95 % highest posterior density (HPD) interval: 1984–1991). The predominantly H3N2 introduction into Europe (B) occurred around 5 years later, with a median TMRCA of 1994 (95 % HPD: 1990–1998).

These multiple introductions of HA and NA segments resulted in a mix of subtypes in the Eurasian swine clade (as defined by the internal protein-coding segments). The detailed subtype composition of the 92 Eurasian swine clade viruses analysed here is shown in Table 2, where the HA and NA types are defined according to major phylogenetic clades (Fig. 2a–d).

**Table 2.** Detailed subtype composition of the 92 Eurasian swine clade polymerase sequences

Detailed subtype	N1-Eurasian swine	N2-Human seasonal
H1-Human seasonal	1	18
H1-Classical swine	4	1
H1-Eurasian swine	55	0
H3-Human seasonal	0	13

### Intra-clade polymerase reassortments

Although the Eurasian swine clade is monophyletic in each of the polymerase genes, it does not necessarily follow that there is no reassortment between the polymerase segments within this clade. To detect intra-clade reassortment among the polymerase segments, we created concatenated sequences from segments 1+2, 1+3 and 2+3 and used the single break point (SBP) and genetic algorithm for recombination detection (GARD) analyses available within HyPhy (Kosakovsky Pond *et al.*, 2006). Table 3 shows the break points and improvement in Akaike Information Criterion (AIC) score detected in each concatenated segment pair. For all pairs and both algorithms, some evidence for a break point was detected near the artificially joined segment boundaries, indicating the possible reassortment of a small number of taxa. Since we could not rule out some reassortment between the polymerase segments, we decided to use only segment 1 (PB2) in our subsequent phylogenetic-based analysis, rather than all three segments concatenated (however, we do not expect the results to be particularly sensitive to the segment chosen due to the similarity of the trees; see Table S4).

**Table 3.** Break points (in nucleotides) and percentage improvement in AIC (dAIC) or small sample AIC (dc-AIC) score compared with concatenated segments using SBP or GARD algorithms from HyPhy

Segment A	Segment B	Actual break point	SBP break	SBP dAIC (%)	GARD break	GARD dc-AIC (%)
1	2	2287	2291	5.8	2292	4.7
1	3	2287	2294	4.4	2295	3.5
2	3	2281	2285	3.8	2286	3.1

### Reassortment of HA and NA onto the polymerase backbone

To investigate the rate of acquiring different HA and NA segments with respect to an internal protein-coding segment, each of the 92 Eurasian swine PB2 sequences in the predominantly European swine clade was labelled with its detailed HA and NA subtype (e.g. H1-Eurasian swine and N1-Eurasian swine, see Table 2), and these subtype labels were used as discrete traits in a beast analysis. Asymmetrical discrete trait models were jointly estimated with phylogenies using: (i) HA only; (ii) NA only; and (iii) joint HA-NA as the discrete traits. Phylogenies coloured by discrete trait can be found in Figs S21–23.

**Fig. 4.**
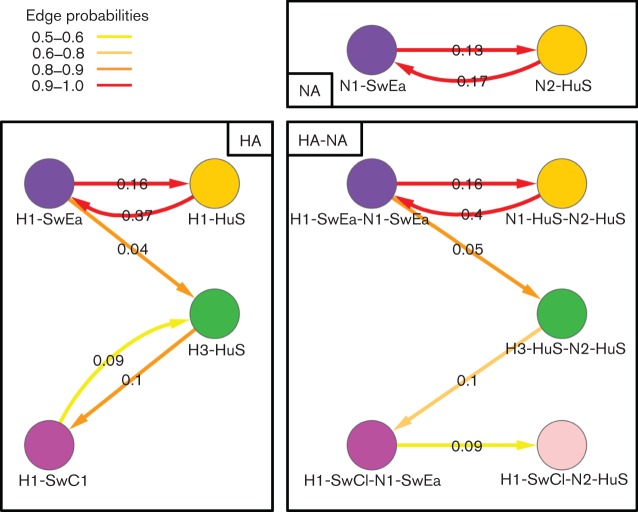
Significant rates for reassortments (expressed as exchanges year^−1^) onto the PB2 backbone, calculated using beast discrete traits. HA only (left), NA only (top right) and HA-NA joint (bottom right) traits results are shown. The detailed subtype abbreviations are as for Fig. 2. Only those edges with a probability of existing in the trees sample ≥0.5 and Bayes factor ≥4 are shown, and the colour reflects the support for the edge ranging from red (strongest) to yellow (just significant). The edge values are median transition rates.

The rates of transition between one state and another were calculated for all state pairs and Bayesian stochastic search variable selection (BSSVS) Bayes factor tests (Lemey *et al.*, 2009) were used to determine which of these rates were significant. Fig. 4 is a network transition diagram showing the significant rates between HA only, NA only and joint HA-NA subtype traits. The colour of the edges depicts the significance of the rate and the values represent the median transition rate per year. Table S4 contains the mean, median, sd and 95 % HPD intervals for all the rates and their mean indicator values. Results for HA on PB1 are also included in Table S4 for comparison.

Considering NA only, the median rate at which Eurasian swine N1s (N1-SwEa) switch to human seasonal-origin N2s (N2-HuS) on the polymerase backbone is 0.13 exchanges year^−1^; conversely the median rate for N2-HuS changing to N1-SwEa is 0.17 year^−1^ which is not significantly different (Table S4).

The most significant rates for HA switching were between Eurasian swine H1s (H1-SwEa) and human seasonal-origin H1s (H1-HuS); the H1-HuS-to-H1-SwEa rate was higher than that for the opposite direction (0.37 compared with 0.16 exchanges year^−1^). Significant but lower switching rates were also found for H1-SwEa and H3-HuS (human seasonal-origin H3), and H3-HuS to and from H1-SwCl (classical swine H1). The rates between the two swine lineages (H1-SwEa and H1-SwCl) and two human seasonal-origin lineages (H1-HuS and H3-HuS) were not significant.

The rates of reassortment for the joint HA-NA labelling show a similar pattern to those for HA only, because the NA-only rates are so similar to each other. The rates for H1-SwEa-N1-SwEa (H1N1 Eurasian swine) to/from H1-HuS-N2-HuS (H1N2) are almost the same as those for H1-SwEa to/from H1-HuS. In particular the rate for H1N2 changing to H1N1 Eurasian was high (0.4 exchanges year^−1^), whilst the reverse rate was significantly lower (0.16 exchanges year^−1^). As with the HA-only case, the rates between swine lineages H1-SwEa-N1-SwEa and H1-SwCl-N1-SwEa, and the two human seasonal-origin lineages (H1-HuS-N2-HuS and H3-HuS-N2-HuS) were not significant.

## Discussion

### Reassortant viruses found but not pandemic genome prior to 2009

In this study we undertook full genome sequencing of a set of archived swine influenza isolates, previously collected as part of routine swine surveillance. As well as the major H1N1, H1N2 and H3N2 strains, we found examples of inter-lineage reassortment including two H3N1 strains. Neither we, nor other recently published swine influenza studies in China and the USA (Nelson *et al.*, 2011; Vijaykrishna *et al.*, 2010, 2011), have reported a swine influenza virus with the same segment constellation as the 2009 H1N1v pandemic strain prior to the onset of the pandemic. This does not mean that the pandemic strain definitely did not originate in swine in Europe, Hong Kong and China, or the USA, but that the current global swine influenza surveillance may be insufficient for direct sampling of the reassortant strain that led to the human pandemic.

### Polymerases acquire HA and NA segments through reassortment

Within the European swine strains, we found evidence of the persistence of the Eurasian lineage internal gene segments, despite the introduction of other swine lineages into Europe. A similar effect has previously been observed in North American swine viruses: the triple-reassortant strain which first emerged in the 1990s (Webby *et al.*, 2000; Zhou *et al.*, 1999) initially comprised avian-like PB2 and PA but human-like PB1, HA-H3 and NA-N2, and classical swine lineage NP, MP and NS. Further reassortments with human, avian and classical swine lineage viruses occurred resulting in the acquisitions of different HA and NA upon the same internal genes set (Karasin *et al.*, 2002, 2006; Lorusso *et al.*, 2011; Olsen *et al.*, 2006; Smith *et al.*, 2009), leading to the concept that the combination of these polymerase segments [the triple-reassortant internal gene (TRIG) cassette; see for example Vincent *et al.* (2008)], was particularly fit in the North American swine population. Recent experimental reassortment studies have also indicated that the TRIG cassette seems to confer a selective advantage (Ma *et al.*, 2010). Therefore like the persistence and preferential transmission of the TRIG cassette, we postulate that the polymerases of the Eurasian swine lineage are also a particularly fit combination for transmission in swine.

The dynamics of human influenza strains appear to be driven by the need to evolve antigenic novelty (antigenic drift) to counteract herd immunity (see for example Garten *et al.*, 2009; Boni, 2008; Russell *et al.*, 2008; Smith *et al.*, 2004), and the lineages of the internal gene segments broadly correspond to the lineages of the HA and NA genes, with very little reassortment between circulating subtypes. However, in the commercial swine herd, there is less pressure to select for antigenic changes in influenza viruses for two main reasons (de Jong *et al.*, 2007): (i) the lifespan of an individual animal reared for pork is generally around 6 months and birth rates are very high, ensuring a high proportion of susceptible individuals in the population at any one time; and (ii) there is no widespread use of swine influenza vaccines in Europe. This means that we would not expect the same evolutionary pressures on HA and NA in swine and humans, and indeed swine H3 viruses exhibited a much lower rate of antigenic drift in HA compared with humans (de Jong *et al.*, 2007; Lorusso *et al.*, 2011). So rather than the correlated lineages in all segments being driven by the HA and NA subtype (as for seasonal influenza viruses in humans, see Figs S1–20), we observed persistence and onward transmission of the polymerase encoding segments over several years in European swine regardless of HA and NA subtype. Therefore, for swine, it is appropriate to consider the polymerase segments as the primary heritable units which acquire different glycoprotein segments through reassortment.

### High reassortment rates between endemic swine lineages

Although influenza infection only lasts a few days within an individual swine (e.g. Brookes *et al.*, 2010), recent work has shown that an influenza strain can persist on a farm for several months by infection of successive groups of susceptible pigs being born on the farm (Williamson *et al.*, 2012). In the UK in 2006–2009, 13 % of farms tested were found to be seropositive for both H1N1 and H1N2 (Mastin *et al.*, 2011). In Spain in 2001–2004, 33 % of herds tested seropositive for at least two subtypes of H1N1, H1N2 and H3N2 (Maldonado *et al.*, 2006), and in 2002–2003 20 % of animals tested in Belgium, Germany and Italy were seropositive for two subtypes (Van Reeth *et al.*, 2008). Consequently the opportunity for reassortment between swine influenza strains is present within the swine industry in Europe.

Previous work has shown how discrete trait mapping can be used to infer the transmission rates between cities (Lemey *et al.*, 2009; May *et al.*, 2011; Raghwani *et al.*, 2011); here we used the same methodology and software to infer the reassortment rates between subtypes/lineages, extending the application of the Bayesian phylogeographical method to quantifying the rate of ‘recombinant chatter’ (Vijaykrishna *et al.*, 2011). We found that the rate of reassortment between the HA and NA from H1N1 and H1N2 subtypes, with respect to PB2, was particularly high, estimated as at least 0.4 events year^−1^ (i.e. a reassortment every 2 or 3 years). The true rate of reassortment in swine is likely to be higher since some reassortant viruses will not have been sampled. This high inter-subtype reassortment rate in swine is similar in magnitude to the intra-subtype reassortment rate indicated in a study of human seasonal H3N2, in which two reassortment events were identified over a period of 5 years (Holmes *et al.*, 2005).

In contrast to the high rates of reassortment between H1-Eurasian and H1-human seasonal-origin HA segments, the rates between H1-Eurasian swine and H1-classical swine were not found to be significant, meaning that transitions of this type were rare in the predominantly European dataset we analysed. Additional isolates with Eurasian PB2 segments would be required to estimate the H1-Eurasian to/from H1-classical reassortment rates and improve upon the H1-classical to H3-human seasonal-origin rates; however, such isolates may be more likely to be found in the recently sequenced Asian samples (Vijaykrishna *et al.*, 2011) than in our European study, especially given that classical lineage H1s have not been observed in European swine since 1993.

We also did not find significant rates of reassortment between H1-human seasonal-origin and H3-human seasonal-origin HA on the Eurasian PB2 backbone. These low and insignificant rates are unexpected, since H1N2 and H3N2 subtypes are known to co-circulate in continental Europe (Maldonado *et al.*, 2006; Van Reeth *et al.*, 2008). However, the Eurasian PB2 tree (Fig. S23) shows a distinct split between Continental European (middle clade) and English (lower clade) sequences. In the English clade there were only two H3N2 sequences from 1993 and 1994 that seem to reassort with the prevailing Eurasian H1N1 lineage. In the continental clade, there is only one direct transition of H1N2 to/from H3N2 and it is not well supported, hence a lack of significance for these transitions in our analysis. The observed pattern can be explained by the separate introductions of mainly H1N2 (clade A in Fig. 2) and H3N2 (clade B in Fig. 2) into an existing population of H1N1 Eurasian viruses, consistent with the lower fraction of animals with antibodies to both H3N2 and H1N2 (Van Reeth *et al.*, 2008). Consequently the significant reassortment rates are predominantly between the H1N1 Eurasian strains and the H1N2 and H3N2 strains individually.

Our results lead us to propose that co-circulation of strains within swine herds leads to frequent inter-subtype reassortment between strains. Furthermore, since transmission of the H1N1v pandemic strain into swine herds has been reported in several countries (Brookes & Brown, 2011; Forgie *et al.*, 2011; Howden *et al.*, 2009), it is not unexpected that subsequent pandemic/endemic reassortments have been detected in swine (Ducatez *et al.*, 2011; Howard *et al.*, 2011; Kitikoon *et al.*, 2011; Moreno *et al.*, 2011; Starick *et al.*, 2011; Tremblay *et al.*, 2011; Vijaykrishna *et al.*, 2010). Assuming that farm biosecurity and vaccination policies remain as they have been in recent years, we should expect that further new reassortant strains will arise and persist in European swine herds over the next few years.

## Methods

### 

#### RNA extraction, PCR methods and sequencing.

Viral RNA was extracted from samples using the QIAamp Viral RNA Mini kit according to the manufacturer’s instructions (Qiagen) and RT-PCR-amplified using an eight-segment PCR method (Zhou *et al.*, 2009), with some modifications as described by Bhatt *et al.* (2012). In particular, three RT-PCRs were performed for each sample using primers common_uni12 (5′-GCCGGAGCTCTGCAGATATCAGCRAAAGCAGG-3′), common_uni12G (5′-GCCGGAGCTCTGCAGATATCAGCGAAAGCAGG-3′) and common_uni13 (5′-GCCGGAGCTCTGCAGATATCAGTAGAAACAAGG-3′). The first and second reactions contained the primers common_uni12 and common_uni13; however, the second RT-PCR was a control PCR in which the reverse transcriptase was omitted and which contained only Platinum *Taq* HiFi polymerase, to exclude the presence of DNA contamination. The third contained the primers common_uni12G and common_uni13 (greatly improving amplification of PB2, PB1 and PA). Prior to sequencing, equal volumes of reactions one and three were combined and DNA concentration was determined using the Quant-iT PicoGreen dsDNA Picogreen assay (Invitrogen).

All samples were sequenced by Illumina sequencing (Illumina); some samples were also sequenced on the 454 platform (454 Life Sciences). RT-PCR products were converted to multiplexed sequencing libraries for Illumina or 454 following the manufacturers’ instructions. For Illumina sequencing, samples were multiplexed up to 12 samples per lane, and were sequenced on the Illumina Genome Analyzer IIx platform with 54 bp paired-end reads. For 454 sequencing, samples were multiplexed up to 12 samples per region of a four-region PicoTiter Plate (Roche 454), and were sequenced using single-end runs on the GS FLX Titanium platform.

#### Sequence mapping and consensus sequence generation.

An important consideration in the sequencing strategy was that we did not know a priori which reference sequences would be most appropriate for each segment of the isolate because of the prevalence of reassortment. We therefore developed a strategy to efficiently select from a set of potential reference sequences.

We obtained all full-length or near full-length influenza sequences from GenBank and filtered them such that no sequence was <6 % different by sequence identity from any other sequence, resulting in a final reference sequence set comprising of between 87 (NP) and 289 (NA) sequences per segment (see supplementary material for reference sequence set).

Mapping was performed in two stages: (i) to identify the most appropriate reference sequence for each segment from the filtered set of reference sequences; and (ii) to map all reads against the best reference sequence set and generate a consensus. Both mapping stages were performed using ssaha2 (Ning *et al.*, 2001) using the ‘-rtype solexa’ option for Illumina reads and the ‘-rtype 454’ option for 454 reads, and the ‘-skip 1’ option for both read types. The first stage was performed using the default ‘-best’ setting, reporting all matches for each read. Following the first stage of mapping, the best reference sequence for each segment was selected by ranking the mapping results by three criteria: (i) percentage of the reference sequence covered by at least 10 high-quality bases; (ii) number of high-quality nucleotides in mapped reads; and (iii) mean coverage. The best reference sequences were then used for the second stage of mapping, which was performed using identical parameters as the first round, except that the ‘-best’ option was set to 1, restricting the reporting of only the best match for each read. Consensus sequences were generated using SAMtools (Li *et al.*, 2009) and our own Perl scripts (available from the authors on request). This procedure allowed accurate sequence determination when the reference sequence was unknown.

Some reference sequences were not full length. To ensure the final consensus sequences extended to the ends, we employed an iterative-mapping strategy using the ssaha2 ‘-output sam_soft’ option, which generates sam (Sequence Alignment/Map) format output including both the matching and the non-matching portions of reads. The overhanging regions of reads mapping to the ends of the existing sequences were used to create consensus sequences, which were appended to the original sequences. This process was repeated until all segments had termini matching the expected influenza A termini.

Finally, the mapping was repeated against the consensus sequences, to create a final consensus sequence.

#### Background sequence data.

The sequences obtained above were analysed in conjunction with a sample of 379 other available full genome sequences obtained from the NCBI Influenza Virus Resource (Bao *et al.*, 2008). The additional data consisted of: (i) one full genome sequence per host species (human, avian, swine), per location (country), per year for H1N1 (non-pandemic), H1N2, H2N2 and H3N2, if available; (ii) three human pandemic H1N1v full genome sequences from each of 2009 and 2010; and (iii) five pandemic H1N1v full genome swine sequences (see Tables 1 and S3).

#### Phylogenies.

Phylogenies for each segment of the background and Combating Swine Influenza Initiative (COSI) sequences were generated using RAxML (Stamatakis, 2006), employing a maximum-likelihood tree search with the GTR+gamma model (general time reversible+gamma distribution for rates over sites) and 1000 bootstraps. For HA and NA, separate trees were estimated according to subtype, i.e. H1, H3 (segment 4) and N1, N2 (segment 6).

#### Molecular clock phylogenies.

Time-resolved trees were generated for each internal protein-coding segment of the complete genome COSI sequences together with the other human, swine and avian background sequences (448 sequences per segment, Table S3) using beast via beauti (version 1.6.1) XML input files (Drummond & Rambaut, 2007). Time-resolved trees were also generated for each subtype of HA and NA separately (i.e. HA-H1, HA-H3, NA-N1, NA-N2).

Exact isolate dates were used in the analysis where possible; but if only the month and year of isolation was known then the day was assumed to be the 15th of the month; if neither the month or day was known then the isolation date was taken to be 2 July (61 % of the 448 complete genomes). For segments 1–6, the SRD06 nucleotide substitution model (Shapiro *et al*., 2006) was used [this allows one Hasegawa, Kishino and Yano (HKY) model for codon positions 1 and 2 and a different HKY model for position 3, and gamma-distributed rates across sites], and for segments 7 and 8 GTR with gamma rates over four categories was employed (SRD06 is inappropriate for segments 7 and 8 due to the overlapping reading frames and frameshifts for M2 and NS2, respectively). A relaxed lognormal clock was chosen to allow potentially different rates for the human, avian and swine lineages, and because it was preferred over a strict clock in a Bayes factor test on PB2 (Bayes factor >20) (Drummond *et al.*, 2006). For the large human, avian and swine dataset we also chose a constant population size to approximate the total viral diversity present over the three distinct lineages (see Fig. S24). For the internal protein-coding segments, the maximum clade credibility (MCC) trees were composed of two independent runs of 50 000 000 Markov chain Monte Carlo (MCMC) steps, initially sampled every 5000 steps, combined with a 10 % burn-in and then further down-sampled to 9000 trees, giving a posterior effective sample size of at least 200. For HA and NA, the MCC trees were composed of five independent runs of 20 000 000 MCMC steps, initially sampling every 1000 steps with a 10 % burn-in and then further down-sampled to 18 000 trees, giving a posterior effective sample size of at least 500.

#### Discrete trait mapping and reassortment rates.

The rates of change from one discrete trait to another can be calculated within beast, by employing models of trait evolution. Additionally Bayesian stochastic search variable selection (BSSVS) was used to determine which rates are statistically significant (Lemey *et al.*, 2009). These methods have been used previously in order to infer movement rates between geographical regions (Lemey *et al.*, 2009; Lycett *et al.*, 2012; Nelson *et al.*, 2011) Here we used the specified subtypes as the discrete traits to be modelled upon the PB2 trees. Asymmetrical models of discrete traits were employed with BSSVS, and beast input XML files were generated using beauti as before. The SRD06 nucleotide substitution model, relaxed lognormal clock model and skyride population model (Minin *et al.*, 2008) were used for the 92 Eurasian PB2 sequence set (see Fig. S25). Two independent runs per model were performed with 50 000 000 MCMC steps, a 10 % burn-in and sampling every 10 000, yielding 9000 tree samples after combining the two runs, and effective sample sizes of over 100 (typically 1000–4000) in all parameters.

#### Reassortment detection.

Reassortment detection between pairs of segments was performed within HyPhy using the single break point and genetic algorithm for recombination detection (gard) standard analyses (Kosakovsky Pond *et al.*, 2006). For both types of analysis a GTR model with four gamma rate categories was used.
